# Emergency Department Discharges Following Falls in Residential Aged Care Residents: A Scoping Review

**DOI:** 10.3390/jcm14145169

**Published:** 2025-07-21

**Authors:** Gigi Guan, Kadison Michel, Charlie Corke, Geetha Ranmuthugala

**Affiliations:** 1 Department of Rural Health, Melbourne Medical School, The University of Melbourne, Shepparton, VIC 3630, Australia; granmuth@une.edu.au; 2 Critical Care Unit, Goulburn Valley Health, Shepparton, VIC 3630, Australia; kadison.michel1@gvhealth.org.au; 3 School of Medicine, Deakin University, Geelong, VIC 3220, Australia; charlie.corke@deakin.edu.au; 4 Critical Care Units, Barwon Health, Geelong, VIC 3220, Australia; 5 School of Rural Medicine, University of New England, Armidale, NSW 2350, Australia

**Keywords:** Advance Care Directive (ACD), emergency department (ED), falls, prehospital care, residential aged care facility (RACF)

## Abstract

**Background:** Falls in residential aged care facilities (RACFs) have a significant impact, often leading to costly and unnecessary emergency department (ED) transfers. This scoping review examined the ED discharge proportions and patient characteristics of RACF residents presenting to the ED following a fall, to identify factors that could reduce unnecessary ED transfers. **Methods:** The databases MEDLINE, CINAHL, Scopus, and Web of Science were searched, resulting in an initial 1385 articles. Nine of these articles met the inclusion criteria and were included in this review. **Results:** The median age of patients reported in the nine papers ranged from 80.8 to 88 years. Discharge proportions from ED back to RACF ranged from 36% to 91%, with an average of 63%. The studies that reported on computed tomography of the brain (CTB) showed that CTB findings did not significantly influence discharge decisions. **Conclusions:** Many RACF residents transferred to EDs following falls are discharged without hospital admission. The heterogeneity of study methods makes it challenging to draw definitive conclusions about factors that may help identify patient groups that do not require transfer to the ED following a fall. However, this scoping review highlights potential opportunities to reduce ED transfers from RCAFs. These findings highlight a need for geriatric-specific, person-centred protocols that reduce unnecessary ED transfers while safeguarding quality of care and respecting residents’ advance care preferences.

## 1. Introduction

Falls in the geriatric population, particularly among residents in residential aged care facilities (RACFs), present a critical concern due to the high risk of injury and subsequent healthcare utilisation [1,2,3]. RACFs, as environments tailored to care for older adults, frequently encounter such incidents, prompting a reassessment of safety protocols, fall prevention strategies, and, more importantly, post-fall management practices. Residents in RACFs often experience frailty, polypharmacy, and cognitive impairment, which increases the risk of falls and complicated decisions regarding ED transfer [4]. Falls in RACFs often necessitate transfers to emergency departments (EDs) for evaluation and management, although such transfers may not always be necessary [5]. ED transfers are often policy-driven, especially when trained personnel, such as general practitioners (GPs) or nurse practitioners (NPs), are unavailable to perform medical reviews at RACFs [4]. Medical review is frequently mandated after a fall in RACFs, regardless of the patient’s presentation, mechanism of injury, or the severity of the fall. The post-event transfer of older adults to EDs often requires either an emergency or non-emergency ambulance, which is costly to the healthcare system [4]. In high-income countries with ageing populations, fall-related ED visits represent a growing public health burden with implications for system sustainability and quality of life [3].

Understandably, prompt medical review is essential to pre-emptively address potential internal injuries or complications that may not be immediately visible. However, this also necessitates careful examination of its implications for individual residents and the broader healthcare system [6]. The impact of such transfers extends beyond immediate clinical outcomes. They also affect residents’ psychological well-being, contribute to potential overuse of emergency healthcare resources, and have broader implications for healthcare policy and RACF care standards.

This scoping review (SR) aimed to investigate the proportion of discharges from the ED back to the RACF following presentation due to a fall. Also explored are factors that could reduce ED transfers of residents from RACFs after a fall. Being able to identify factors associated with being discharged from the ED back to RACFs has the potential to enhance the quality of care for older adults in RACFs, whilst also helping reduce costs to the health system.

## 2. Methods

### 2.1. Study Design

The methodology followed guidelines from the Joanna Briggs Institute and adhered to the framework proposed by Arksey and O’Malley [7]. The review followed five key stages: (1) defining and refining the research question; (2) identifying relevant studies; (3) study selection; (4) charting the data; and (5) collating, summarising, and reporting results. This approach is well-suited for exploratory topics with high methodological heterogeneity and was selected over a systematic review due to the wide variation in outcomes, definitions (e.g., ‘avoidable ED transfer’), and patient characteristics in the existing literature. This review also followed the PRISMA-ScR (Preferred Reporting Items for Systematic Reviews and Meta-Analyses extension for Scoping Reviews) checklist to promote transparency and reproducibility. No protocol was registered for this SR.

In this SR, discharge proportions were used rather than terms such as preventable or avoidable presentations to the ED. This is because there is no universal consensus on what constitutes preventable or avoidable presentations [8,9]. The research question, developed according to the Population, Intervention, Comparison, and Outcome (PICO) guidelines, was used to identify potential studies. Ethical approval was not obtained as direct patient involvement was not required.

### 2.2. Search Strategy

A comprehensive search was conducted across electronic databases, including MEDLINE, CINAHL, Scopus, and Web of Science on 3 March 2024. The keywords ‘falls,’ ‘residential aged care facilities,’ and ‘emergency department’ were used in the search. The Boolean operators ‘AND’ and ‘OR’ were used to combine the search terms. All potential articles for review were entered into Covidence, a systematic review screening software, for initial screening and data extraction (Appendix A, search strategy).

Two authors (GG and KM) independently screened all the articles based on article titles and abstracts. Any disagreements or conflicts were resolved by consensus. Inter-rater reliability was calculated using Cohen’s Kappa generated by Covidence, with a substantial agreement for title and abstract screening (κ = 0.65) and almost perfect agreement for full-text review (κ = 0.94).

### 2.3. Eligibility Criteria

Studies were eligible for inclusion in the SR if they met the following criteria: the primary focus was on falls from standing height, mechanical falls, falls due to trips, or accidental falls. Eligible studies were limited to those involving human participants residing in RACFs. Only full-text articles published in English between 1 January 2010 and 1 March 2024 were considered. Both systematic reviews and primary studies conducted in any country were included. The following exclusion criteria were applied: studies primarily reporting on fall prevention interventions or programmes; falls related to motor vehicle incidents or animal interactions; condition-specific presentations such as those associated with drug or alcohol use, or dementia; and non-systematic literature reviews, case reports, or opinion pieces. In addition, discharge data not reported or that could not be calculated were also excluded.

The exclusion of studies with alcohol/medication/dementia-related conditions was intentional. This is due to the SR’s aim to focus on undifferentiated fall-related presentations, where ED transfer decisions may not be clear [10]. Falls from RACFs that are due to clear causes, such as medication, dementia related conditions, are often required to be transferred to the ED for assessment and ongoing care [4]. In contrast, falls with no apparent injuries or clearly defined causes can often lead to an ambiguous decision-making process for ED transfer due to uncertainty in post-fall care [10].

### 2.4. Data Processing and Analysis

Data was extracted using Covidence, with emphasis on the following variables: participant demographics (e.g., age and sex); the reported number of admissions to ED from RACFs due to falls; the proportion of ED presentations that resulted in discharge back to the RACFs; interventions provided during the ED stay; and the status of advance care directives (ACDs) if recorded.

## 3. Results

**Figure 1:**
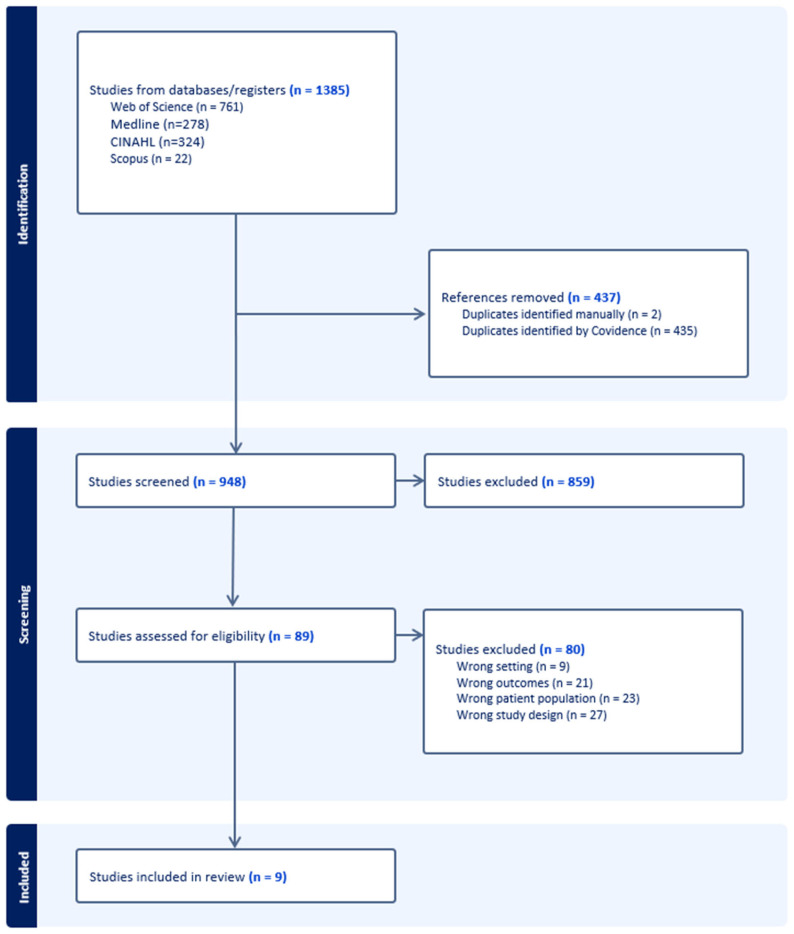
PRISMA-ScR flow diagram.

The initial search yielded 1385 articles from four electronic databases. After removing 437 duplicates using Covidence, 948 articles remained for title and abstract screening. Of these, 859 were excluded due to irrelevance, leaving 89 articles for full-text review (Figure 1). Nine of these met the inclusion criteria and were included in the final SR. Eighty articles were excluded for incorrect study design (*n* = 27), incorrect patient population (*n* = 23), incorrect outcomes (*n* = 21), and incorrect settings (*n* = 9).

### 3.1. Characteristics of the Included Studies

Five out of nine studies reviewed were conducted in Australia [11,12,13,14,15]. The rest were from Spain [16], Korea [17], Ireland [18], and Sweden [19]. The study period ranged from 2011 to 2023. The median age of the patients reported in these studies ranged from 80.8 to 88 years. All studies used a retrospective review of medical records to gather data (Table 1).

### 3.2. Discharge Proportions from ED Post-Fall in RACF Patients

The total number of fall-related ED transfers from RACFs from the nine studies reviewed was 10,824, of which 4932 (46%) were discharged from the ED back to RACFs. The individual study discharge proportions from the ED varied significantly, ranging from 36% to 91%, with an average discharge proportion of 63%. Studies conducted in Australia generally reported higher discharge proportions, with Green et al. and Tulchinsky et al. reporting 91% and 79% discharge back to RACFs, respectively [12,15]. In contrast, Choi et al. in Korea reported a discharge proportion of 36% [17]. Intermediate proportions were reported in studies from Spain (55%) and Sweden (61%) [16,19].

### 3.3. ACD and CTB Post-Fall from RACFs

While all studies examined discharge proportions, only a few reported ACD or CTB status during ED visits. Green et al. reported that 80.3% of the 366 patients had CTBs, with 6% (*n* = 22) showing abnormal intracranial pathology, and only one patient (0.3%) required reversal of anticoagulation [12]. Similar findings were reported in the study conducted by Tulchinsky et al., which showed that out of 569 fall-related patient presentations from RACFs, 538 (69%) had CTB, of which 31 (6%) demonstrated abnormal intracranial pathology. No patients received neurosurgical interventions in either study [15]. In the included studies, the documentation of ACDs was limited, with only three studies addressing this aspect. The percentage of patients with an ACD ranged from 50% to 63% [13,14,15].

## 4. Discussion

Falls in RACFs often result in ED transfers, although many residents are discharged back to RACFs without requiring hospital admission or significant intervention. A recent study indicates that nearly 70% of undifferentiated post-fall RACF patients were discharged from the ED [10]. The study results suggest opportunities to manage some cases within RACFs, potentially reducing healthcare resource utilisation and better aligning with patient preferences. Such opportunities include fall prevention [20], post-fall care referral pathways [21] and on-site post-fall monitoring [10]. All of the above should not only aim to reduce ED transfers but also provide patient-centred care and ensure ongoing patient safety.

Frequent ED transfers and subsequent hospitalisation of frail older adults have been associated with adverse outcomes, including delirium, functional decline, and hospital-acquired complications such as infections and iatrogenic injury [22]. These risks are especially concerning in the RACF population, where many residents live with multiple comorbidities, cognitive impairment, and reduced physiological reserve [4]. Avoiding unnecessary ED transfers may help preserve baseline function, reduce stress and confusion, and maintain quality of life [22]. Moreover, the unfamiliar and overstimulating ED environment may contribute to hospital-induced delirium, which is linked to longer-term cognitive decline and institutionalisation [23]. These considerations further support the need to develop protocols that enable appropriate in-place management for undifferentiated fall cases, particularly when aligned with residents’ ACDs.

### 4.1. Reasons to Transfer to ED Post-Fall from RACFs

From the RACFs’ perspective, the decision to transfer a resident to the ED after a fall involves multiple factors, including the availability of on-site medical or other staff trained to assess the resident following a fall, the severity of the injury, time of day, and day of the week [24]. However, the decision to transfer patients to the ED is often protocol- and policy-driven, especially for those on anticoagulant or antiplatelet medications, which represents a large proportion of the RACFs population [24].

Guidelines or protocols used in post-fall care in Australia often mandate a complex schedule of vital sign monitoring, up to 20 observations over 48 h, to ensure patient safety and detect any deterioration promptly in the State of Victoria and Queensland [10]. However, RACFs often face resource limitations that make it challenging to adhere to these intensive observation schedules. As a result, the decision to transfer post-fall patients to the ED for medical evaluation became necessary, notably after hours or during weekends, when access to GPs or NPs may be limited.

RACF patients often use antiplatelet or anticoagulation due to advanced age and multiple comorbidities. This may increase concerns for potential intracranial pathology after a fall. However, numerous studies have reported that antiplatelet and/or anticoagulant agents do not increase the risk of intracranial bleeding, particularly when falling from a standing height compared to patients not taking such medications [25,26,27]. This is also supported by two articles included in this review. Of the total of 935 patients who had a fall from RACFs, 832 (89%) underwent CTB scans, of which only one patient required anticoagulation reversal (0.11%) and no patient required neurosurgery, as reported in either study [12,15]. Both studies have demonstrated that the outcome of the CTB did not influence post-fall discharge decisions. These findings suggest the need to examine further the evidence supporting routine ED transfers for CTB in the absence of clinical indications. While this review found that CTB outcomes rarely altered immediate management, especially in frail residents, it is essential to acknowledge that post-CT observation is a critical precaution.

A high proportion of post-fall patients being returned to RACFs without any hospital admissions may suggest opportunities to manage some patients within RACFs. Strengthening RACFs’ capabilities to assess and monitor residents after a fall requires improved access to on-site medical assessments, enhanced staff training on post-fall protocols, and sufficient resources for monitoring.

### 4.2. Respecting Patients’ Wishes: A Collaborative Approach for ED Transfers

In this SR, the status of ACD was only reported in three studies, indicating a low level of documentation for this patient group [13,14,15]. The importance of ACD’s presence is evident in its ability to provide patient-centred care [28]. Thus, the lack of documented ACDs may have contributed to the unnecessary transfer to the ED following a fall. The decisions to transfer to the ED post-fall often err on the side of caution, even when hospital care may not be necessary or in the patient’s best interest. Moreover, the included studies found that ACDs were often documented unclearly or ambiguously, leaving room for interpretation by healthcare providers. This frequently results in hospital transfers, even when the patient’s wishes might not have supported such a decision [13].

In the study conducted by Gullick et al., 26% (*n* = 73) of 285 patients were transferred to the ED against existing ACD [13]. Despite the clear documentation of patients’ wishes, clinical decisions frequently overrode these preferences. Similarly, Tulchinsky et al. found that 77% (*n* = 24) of the 31 residents presenting to the ED with acute trauma had an ACD, indicating no active treatment should be pursued, again suggesting that transfer to the ED should have been avoided for these residents [15]. These highlights significant discrepancies between patient preferences and clinical actions, underscoring the need for more stringent adherence to ACDs in healthcare decision-making.

Healthcare professionals should respect and not override patients’ wishes regarding active management, including ED transfers. Engagement with the patient, and when possible, family members or caregivers, in the decision-making process, and understanding their preferences and wishes is essential in patient-centred care [10]. This collaborative approach ensures that the patient’s preferences, values, and desires are taken into account in the transfer decision [21]. Discussing the potential risks and benefits of ED transfers with patients or caregivers helps align decisions with the patient’s expressed wishes or best interests [29].

### 4.3. High Study Heterogeneity

High study heterogeneity was observed in the studies reviewed in this SR, especially in the data reported; much of the emphasis was on reporting discharge proportions along with preventable or avoidable presentations to the ED. The studies varied in their definitions, methodologies, and outcomes measured, which limits the generalisability of the findings. Several studies did not describe or set criteria to define what constitutes preventable or avoidable presentations in the ED. While these may be universally accepted terms [8], using ambiguous definitions leads to difficulties in synthesising research outcomes. In addition, the inconsistent reporting of investigations and management in the included studies makes it challenging to compare or synthesise research outcomes effectively. Specifically, variations in the reporting of ED interventions and management approaches hinder a clear understanding of the characteristics of RACF residents discharged after a fall.

Furthermore, the lack of consistent reporting on co-morbidities limits the ability to contextualise the findings and identify patient-specific factors that may influence discharge decisions. Examining interventions and management provided by the ED can demonstrate that transfers may often not require ED-level management [30]. This can lead to further policy development for managing post-fall patients within RACFs. Furthermore, it is also essential to recognise that falls in RACFs represent a heterogeneous group, with varying underlying causes and patient conditions. Falls may be due to frailty, cognitive impairment, vision impairment, medication side effects, or a chronic disease that affects mobility. Grouping all falls together may overlook important differences [3]. Therefore, future research should consider stratifying falls based on severity, cause, and patient characteristics to provide more nuanced insights.

### 4.4. Strengths and Limitations

This SR provides an overview of current published research on fall-related presentations by aged care residents to ED. The characteristics, discharge proportions, and patient outcomes were included, which offered further research directions. However, SRs have certain limitations. While an SR is effective in identifying broad themes and gaps, compared with a systematic review, SRs do not assess the quality of studies and are therefore unable to make definitive conclusions on the effectiveness of different interventions or practices (such as the transfer to ED following a fall). Additionally, this review included only nine studies, with a significant proportion originating from Australia. This may introduce selection bias due to the search terms used, limiting the applicability of the findings to other countries.

Additionally, this study did not include falls related to a specific cause, such as alcohol consumption, medication use, or any particular medical condition. Furthermore, specific injuries sustained after the fall, such as clear fractures and apparent injuries, were also excluded. Understandably, falls with a specific cause or injury sustained from a fall are indicative of ED transfer. However, not all post-fall patients in the RACF population require hospital admission despite a known cause or apparent injuries. Thus, omitting such studies might limit the proportion of the RACF post-fall population.

All studies included in this SR are retrospective analyses of medical records—a design constrained by reliance on existing data without access to additional information necessary for effective comparison and contrast among studies. There was no consistency in the information collected. This, together with incomplete reporting on comorbidities or interventions, limited the comparative analysis.

An additional limitation is that several conclusions in the discussion are supported by the external clinical literature and national guidelines, which were not part of the original scoping search. While this was necessary for contextualising limited data, it introduces interpretive risk and should be addressed in future studies with a broader scope.

## 5. Conclusions

This SR reveals that a substantial proportion of older adults from RACFs were transferred to EDs following falls and subsequently discharged back to their facilities. The high discharge rates, particularly in the Australian setting, indicate that many of these patients might not require the advanced care available in EDs and could be managed safely within RACFs. Frequent transfers to EDs post-fall were often policy-driven and also due to resource limitations within RACFs. The findings indicate the need for developing and implementing practical guidelines that enable appropriate management of residents within RACFs. Future post-fall management strategies should align more closely with patients’ goals, leveraging patient-centred approaches that combine medical appropriateness, systemic capacity, and patient preferences.

## Figures and Tables

**Table 1:** Characteristics of the included studies.

First Author, Year, Country	Total Number of Participants	Number of Falls	Number (%) of Falls Discharged	Participant Age Mean (SD)	ACD Status	CTB Status
Afonso-Argilés et al., 2023, Spain [16]	1982 patients (2444 presentations)	1384	768 (55%)	85.9 (7.2)	Not mentioned	Not mentioned
Choi et al., 2023, Korea [17]	14,469	7193	2576 (36%)	80.8 (7.3)	Not mentioned	Not mentioned
Close et al., 2011, Australia [11]	18,902 presentations	589	324 (55%)	80.8 (6.7)	Not mentioned	Not mentioned
Fan et al., 2015, Ireland [18]	465 patients (802 presentations)	126	63 (50%)	82.7 (7.4)	Not mentioned	Not mentioned
Green et al., 2020, Australia [12]	366	366	332 (91%)	86 (IQR 81–91)	Not mentioned	80.3% had CTB scans
Gullick et al., 2022, Australia [13]	448	143	98 (69%)	84	ACD documented in 63%	Not mentioned
Kirsebom et al., 2013, Sweden [19]	594	147	91 (61%)	87 (7.2)	Not mentioned	Not mentioned
Tulchinsky et al., 2023, Australia [15]	784	721	569 (79%)	88 (IQR 83–93)	ACD documented in 50%	68.6% had CTB scans
Uthuman et al., 2023, Australia [14]	310 patients (467 presentations)	155	111 (72%)	84.5 (9.1)	ACD documented in 57%	Not mentioned

ACD, advance care directive; CTB, computed tomography of the brain; IQR, interquartile range; SD, standard deviation. Studies reporting numbers of presentations indicate that multiple presentations occurred for the same patients.

## Appendix A. Search Terms

nursing home* OR Residential aged care* OR Assisted living* OR Long-term care facilit* OR Convalescent home* OR Retirement home* OR Care home* OR Memory care facilit*
Fall* OR “Mechanical fall*” OR “Accidental fall*” OR “Fall from standing height” OR “Traumatic fall*” OR “Fall from chair”
“Emergency care*” OR “Trauma care*” OR “Acute care*” OR “Emergency department*” OR “A and E*” OR “Accident and emergency*”
Older patient* OR Geriatric* OR Elder* OR Aged OR Advanced age* OR Frail elderly* OR Older adult*
